# Using Dynamic Structural Equation Modeling to Examine Between- and Within-Persons Factor Structure of the DASS-21

**DOI:** 10.1177/10731911221137541

**Published:** 2022-12-08

**Authors:** Melissa H. Bond, Robert E. Wickham

**Affiliations:** 1 University of California, San Francisco, USA; 2 Northern Arizona University, Flagstaff, USA

**Keywords:** dynamic SEM, factor analysis, multilevel, time series, psychometrics

## Abstract

The recent integration of traditional time series analysis and confirmatory factor analysis techniques allows researchers to evaluate the psychometric properties of measurement instruments at between- and within-persons levels while accounting for autoregressive dependencies. The current study applies a dynamic structural equation modeling (SEM) latent factor analysis (i.e., DSEM-CFA) to a sample of 333 individuals who completed the DASS-21 at their regular therapy sessions. The results of the DSEM-CFA illuminate the reliability, invariance, and structural features of each DASS-21 subscale both between and within persons. The results suggest that the DASS-21 reliably measures depression, anxiety, and stress symptoms when evaluating differences between persons, but does not reliably assess within-persons fluctuations in symptoms over time. The results also suggest that currently accepted methods of modeling sensitivity to change within an instrument are likely lacking and the DSEM-CFA provides insight into reliability within and between persons, which is extremely important for instruments used across time.

Behavioral scientists and clinical practitioners are often interested in evaluating change in attributes over time; however, the state-trait literature suggests that both stable (trait) and fluctuating (state) elements are present in any measurement, even in instruments that aim to isolate one or the other (Hamaker et al., 2007; Steyer et al., 1999). The traditional confirmatory factor analysis (CFA; Jöreskog, 1969) model, which has dominated psychometric literature for more than half a century, illuminates an instrument’s reliability in detecting differences between individuals, although it reveals nothing of its reliability in detecting an individual’s level of change over time. In addition, several models have been proposed to directly examine reliability within persons, such as Cattell’s (1946) p-technique, and dynamic factor analysis (DFA) model (Molenaar, 1985). These approaches were instrumental in highlighting the importance of evaluating within-persons reliability, and they introduced key concepts, like autoregression and innovation variance, into within-person psychometric analysis (Molenaar, 1985). In the standard DFA model, both the concurrent (*t*) and lagged (*t –* 1) factor structures are modeled, and the concurrent latent factor is regressed onto the lagged version of itself, thereby explicitly incorporating the relationship among repeated observations through the autoregressive parameter (φ; Molenaar, 1985). Although DFA can be applied to *N* > 1 data, evaluating between-persons differences in the time series parameters requires a two-step approach, where *N* = 1 analyses are pooled to provide between-persons parameters.

The recent emergence of an integrated multivariate (i.e., structural equation modeling [SEM]) and multilevel (i.e., mixed effects) modeling framework, under the banner of Multilevel SEM (Mehta & Neale, 2005; B. Muthén & Asparouhov, 2011), provides researchers with the ability to examine measurement models across multiple levels of analysis. This research supports the need for psychometric methods that can model factor structures and reliability at the between and within levels, simultaneously. Although Mehta and Neale’s (2005) multilevel CFA (ML-CFA) accommodates simultaneous within- and between-level factor structures, it is most suitable for cross-sectional clustered data (e.g., patients in a clinic, children in a classroom) because its ability to account for longitudinal data is limited. Specifically, modeling lagged effects within multilevel SEM would require equally spaced time intervals. More recently, Asparouhov et al. (2018) introduced a Dynamic SEM (DSEM; Asparouhov et al., 2018) approach, which integrates SEM, multilevel modeling, and time-series modeling into a unified framework. When applied to intensive longitudinal data, DSEM provides a comprehensive picture of the construct of interest by decomposing a measured outcome into its between-persons and within-persons components. The within-persons level of the DSEM includes the time-specific measurements and the autoregressive relationship between individually varying timepoints. The between-persons level contains the stable (trait-like) component of the outcome, along with the random effects of the autoregressive parameter(s) and the dynamic error term (i.e., innovation variance). Examples of univariate and cross-classified versions of DSEM are emerging in the literature (Hamaker et al., 2018; McNeish et al., 2021). Specifically, McNeish and colleagues (2021) provide an example of using a DSEM framework to embed measurement models within larger time-series models to reduce bias that occurs when using mean scores on instruments. Although McNeish and colleagues (2021) demonstrate a factor analytic extension of DSEM, the use of these models to evaluate the reliability and structure of an instrument, both between and within persons, while accounting for interdependence of adjacent timepoints, has yet to be explored.

## A Dynamic SEM–Confirmatory Factor Analysis

**Figure 1.**
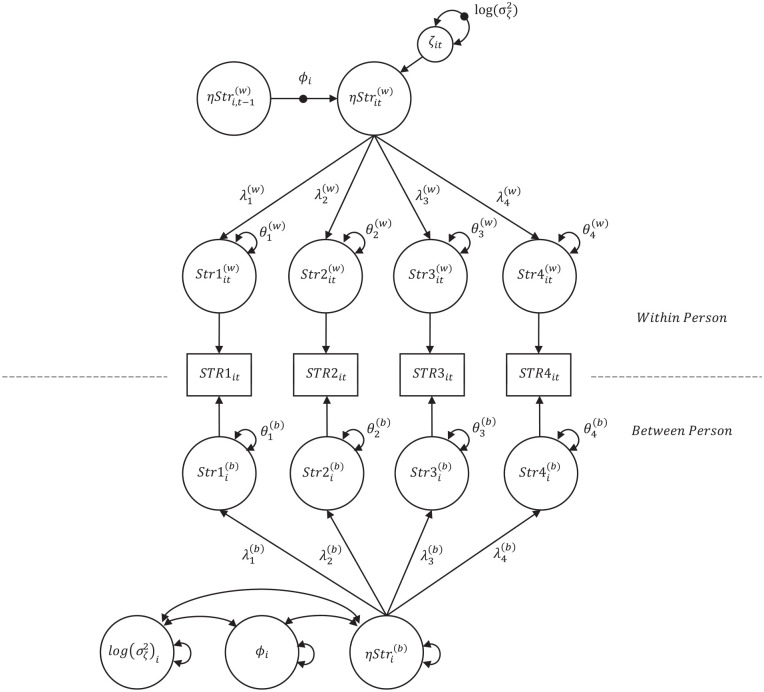
A Two-Level DSEM-CFA of Stress Using Four Indicators *Note.* DSEM-CFA = Dynamic SEM–Confirmatory Factor Analysis; *STR1_it_ – STR4_it_* = observed score for indicator provided by person *i* at time *t; Str1_it_*^(w)^–*Str4_it_*^(w)^ = deviation of indicator for person *i* at time *t; Str1_i_*^(b)^–*Str4_i_*^(b)^ = mean of indicator for person *i*; λ_1_^(w)^–λ_4_^(w)^ = within-level factor loadings; λ_1_^(b)^–λ_4_^(b)^ = between-level factor loadings; η*Str_it_*^(w)^ = within-level latent score for person *i* at time *t*; η*Str_i,t −_*_1_^(w)^ = within-level latent score for person *i* at time *t* − 1; φ_*i*_ = autoregressive parameter for person *i*; ζ_*it*_^(w)^ = innovation variance; η*Str_i_*^(b)^ = between-level latent score for person *i*.

The current study describes the application of a Dynamic SEM to evaluate the between- and within-persons factor structure of a previously validated measure of clinical symptomology among individuals engaged in psychotherapy. The proposed model for a single factor Dynamic SEM–Confirmatory Factor Analysis (DSEM-CFA) is illustrated in Figure 1, using a hypothetical four-item measure of psychological stress as an example. The model decomposes the variance of each observed indicator (e.g., *STR1_it_*) into a within-persons time-specific deviation (i.e., *Str1_it_*^(w)^) and a person-specific mean score for each item (i.e., *Str1_i_*^(b)^). Separate factor loadings and residual variances are specified at the between (λ_(*b*)_, θ_(*b*)_) and within (λ_(*w*)_, θ_(*w*)_) levels. The within-level stress (η*Str_it_*^(w)^) factor is regressed on the stress factor at the previous measurement occasion (η*Str_i_*_,*t* − 1_^(w)^) and the strength of this autoregressive relationship is described by the carryover (φ_*ι*_) parameter. The within-person latent residuals (ζ_it_) are summarized by the innovation variance (σ_ζ_^2^), which follows a log-normal distribution to ensure positive variance during estimation (Asparouhov et al., 2018). In this example, both the carryover and innovation variance parameters are treated as random effects (indicated by the black dot in Figure 1), allowing them to vary across persons. Both random effects are represented as latent variables (φ_*ι*_; log(σ_ζ_^2^)) at the between-persons level and are allowed to covary with one another as well as with the between-persons latent variable of stress (ηStri(b)).

**Figure 2.**
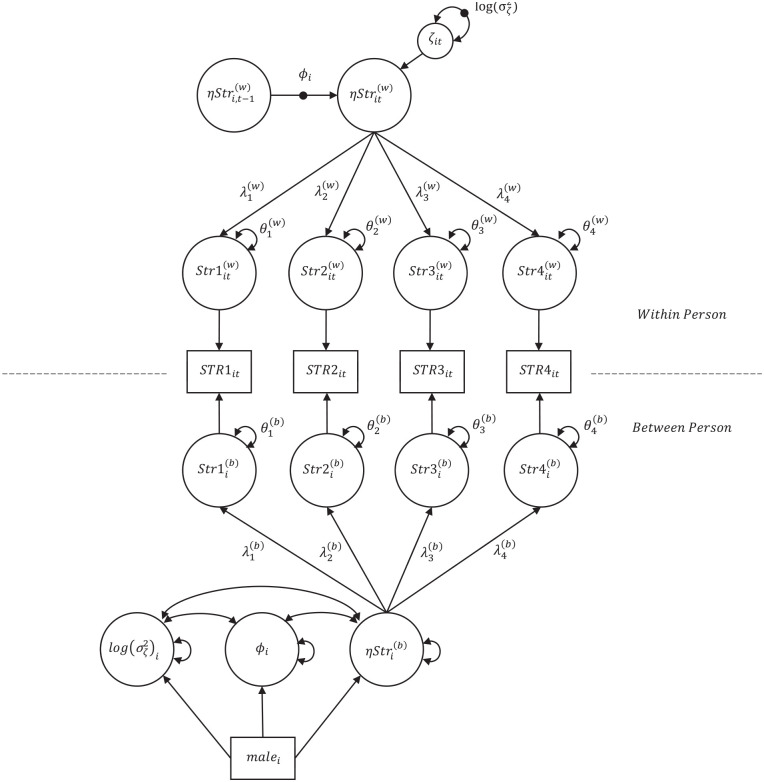
A Two-Level DSEM-CFA of Stress Using Four Indicators, With Gender Included as a Covariate *Note.* DSEM—CFA = A Dynamic SEM—Confirmatory Factor Analysis.

A DSEM-CFA also illuminates the share of variance attributable to state and trait differences, illustrating how an instrument captures differences between persons versus change over time within persons. In addition, the structural model results of a DSEM-CFA provide insight into the strength of the relationship between time points, the random disturbances in scores, and any between-person differences in these parameters. Covariates may also be added to a conditional DSEM-CFA, which may evidence differences in carryover or innovation as a result of person-specific predictors, such as race or gender (see Figure 2).

## Current Study

This study aims to provide a concrete and accessible example of how DSEM may be used to establish the reliability of an instrument used in longitudinal research while accounting for the dependency between adjacent time points. In the present sample, a DSEM-CFA was applied to repeated measurements of the Depression Anxiety Stress Scale (DASS-21; Lovibond & Lovibond, 1995), a commonly used instrument that measures three factors: depression, anxiety, and stress. The DASS was completed by participants every session during active treatment, which provides a real-world example in which symptom change is often measured. The impact of gender on the structural parameters within the DSEM-CFA (i.e., between-level latent factor, carryover, and innovation variance) is also explored, as an example of how covariates may be utilized within this model. The current study emphasizes the importance of utilizing instruments that are reliable within and between persons in longitudinal research and presents an applied example of how DSEM-CFA may be used to achieve this goal.

## Method

### Participants

A total of 333 participants (*M*_age_ = 35.91, *SD*_age_ = 13.8) receiving therapy at a community mental health training clinic were included in this study. Only adult individual clients who completed measures via tablet administration for at least two therapy sessions after the initial intake were included. The sample was majority female (64.3%) and racially diverse (46.4% White, 19.3% Hispanic/Latino, 15.4% Asian, 4.5% Indian/Middle Eastern, 3% Black, 11.4% Other/Mixed). There was an average of 10.76 (*SD* = 6.3) observations per participant via tablet administration of measures.^1^ On average, participants’ scores were in the mild range for depression and anxiety, and in the normal range for stress.

### Measures

Client demographics already present in the clinic database included race/ethnicity, gender, and age, among other variables. Clients were expected to compete the Depression Anxiety Stress Scale (DASS-42, Lovibond & Lovibond, 1995) via tablet administration at the beginning of every therapy session, which occur weekly. The DASS-42 is a 42-item measure with three factors: depression, anxiety, and stress. Given the absence of prior research utilizing this approach, it is prudent to begin with a less complex model. Therefore, only items included within the DASS-21 were included in the present analysis. For the DASS-21, the depression subscale is composed of Items 3, 5, 10, 13, 16, 17, and 21 and includes items such as “I couldn’t seem to experience any positive feeling at all.” The anxiety subscale is composed of Items 2, 4, 7, 9, 15,19, and 20 and includes items such as “I felt I was close to panic.” The stress subscale is composed of Items 1, 6, 8, 11, 12, 14, and 18 and includes items such as “I found myself getting agitated.” The items are rated on a 4-point Likert-type scale from 0 to 3. The DASS-21 exhibits very high internal consistency (all αs above .87; Antony et al., 1998). Test–retest reliability and construct validity across various studies have also been reported as adequate (Antony et al., 1998; Brown et al., 1997; Lovibond & Lovibond, 1995).

### Procedure

No manipulations or experimental conditions were required for the present study. All clients at the clinic are asked to sign an informed consent at the intake session, which includes the possibility that their data will be used in research. The existing data collection protocol at the clinic required that all clients complete the DASS every session.

### Statistical Analysis

The factor structure of the DASS-21 was examined via a DSEM latent factor analysis model^2^ using *Mplus* 8.3 (Muthén & Muthén, 2017). Each seven-item factor of the DASS-21 (depression, anxiety, and stress) was subjected to the DSEM-CFA model described above (see Figure 1). Although previous research on the DASS-21 suggests that the correlations between factors are likely to be nontrivial (Lovibond & Lovibond, 1995), a multifactor DSEM analysis would introduce many new parameters on both the within and between person levels of the model. If a three-factor DASS model were to be specified using DSEM, all random effects at the between level would need to be allowed to covary, resulting in 9 latent factors and 36 covariances. In addition, the relationships between the state latent factors would likely need to be modeled, using a latent cross-lagged panel model, which would also necessitate the addition of innovation covariances (as modeled in Hamaker et al., 2018). Although the estimator used in DSEM allows for complex models with a large number of random effects (Asparouhov & Muthén, 2021; B. Muthén & Asparouhov, 2012), particularly when the researcher incorporates informative priors, the new addition of so many parameters would make the present model unwieldy and difficult to interpret. Therefore, the factor structure of each individual factor will be estimated in the current study to evidence the feasibility of DSEM for factor analysis.

Each seven-item subscale of the DASS-21 (Lovibond & Lovibond, 1995) was examined via a DSEM latent factor analysis model (DSEM-CFA; see Figure 1). For a two-level DSEM-CFA, the model decomposes observed responses to indicators into person-specific (between persons) and time-specific components (within persons). Using the latent factor of stress as an example, the equation for a single indicator can be structured as

(1) $$STR_{it}^{k}=Str_{i}^{k\left(b\right)}+Str_{it}^{k\left(w\right)},$$

where *STR^k^_it_* is the observed score for indicator *k* provided by person *i* at time *t*. *STR^k^_it_* is the mean of the indicator across all measurement occasions for person *i*, and $Str_{it}^{k(w)}$ is the deviation of indicator *k* for person *i* at time *t* from the mean score for that indicator and person, $Str_{i}^{k(b)}.$ The within-level measurement equations further deconstruct the within-persons $\left(Str_{it}^{k(w)}\right)$ variability into components that can be explained by a common factor (i.e., reliable, true score variance), and time-specific residuals representing measurement error and systematic indicator-specific variance:

(2) $$Str_{it}^{k\left(w\right)}=\lambda^{k\left(w\right)}\times \eta Str_{it}^{\left(w\right)}+\varepsilon_{it}^{k\left(w\right)},$$

(3) $$\varepsilon_{it}^{k\left(w\right)}\sim MVN\left(0,\Theta \right),$$

where $\eta Str_{it}^{\left(w\right)}$ is the latent score for within-level stress for person *i* at time *t*, $\lambda^{k(w)}$ represents the within-level factor loading for stress indicator *k*, and $\varepsilon_{it}^{k(w)}$ is the indicator-specific error for person *i* at time *t*. As seen in Equation 3, the $\varepsilon_{it}^{k(w)}$ residuals are assumed to be distributed multivariate normal, with mean 0 and covariance matrix Θ^(w)^. The autoregressive or within-level carryover effect of stress is modeled through a structural latent-on-latent regression described by the following equation:

(4) $$\eta Str_{it}^{\left(w\right)}=\phi_{i}\times \eta Str_{i,t-1}^{\left(w\right)}+\zeta_{it}^{\left(w\right)},$$

(5) $$\zeta_{it}^{\left(w\right)}\sim N\left(0,\sigma_{\zeta ,i\;}^{2}\right),$$

where $\eta Str_{it}^{\left(w\right)}$ is the within-level latent stress score for person *i* at time *t*, $\eta Str_{i,t-1}^{\left(w\right)}$ represents the latent stress score at the previous measurement occasion, and $\phi_{i}$ represents the autoregressive parameter for person *i*. Finally, $\zeta_{it}^{\left(w\right)}$ is the innovation variance, which is assumed to be normally distributed with mean 0 and variance $\sigma_{z,i}^{2}$ .

The between-persons equation for a single indicator can be summarized as

(6) $$Str_{i}^{k\left(b\right)}=v^{k}+\lambda^{k\left(b\right)}\times \eta Str_{i}^{\left(b\right)}+\varepsilon_{i}^{k(b)},$$

where v is the intercept, η*Str_i_*^(b)^ is the latent stress score at the between level for person *i* across all measurement occasions, λ^(b)^ represents the factor loading at the between-persons level, and ε_*i*_^k(b)^ is the between-persons residual. In this model, the within-persons factor loadings λ^(w)^ and between-persons factor loadings λ^(b)^ are allowed to differ for the same indicator, and the present study will estimate the posterior distributions describing the differences in the within- and between-level unstandardized factor loadings. The innovation variance and autoregressive parameters are treated as random at the between-persons level, allowing them to vary across persons as the between-level latent factor does. The equations for these variables can be represented as

(7) $$\left[\begin{array}{c} \eta Str_{i}^{\left(b\right)}\\ \phi_{i}\\ \log\left(\sigma_{\zeta ,i}^{2}\right) \end{array}\right]=\left[\begin{array}{c} \gamma_{\eta Str_{i}^{\left(b\right)}}\\ \gamma_{\phi }\\ \gamma_{\log Var} \end{array}\right]+\left[\begin{array}{c} \mu_{\eta Str_{i}^{\left(b\right)},i}\\ \mu_{\phi ,i}\\ \mu_{\log Var,i} \end{array}\right],$$

where the leftmost vector represents the person-specific parameter estimates for between-level stress (η*Str_i_*^(b)^), carryover (φ_*i*_), and the log of the innovation variance (log $\left[\sigma_{\zeta ,i}^{2}\right]$ ). Each of these parameters is decomposed into fixed (γ) and random components. The fixed components in the middle vector represent the grand mean for each parameter (e.g., grand mean of between-level stress for all persons) and the random components in the rightmost vector represent the person-specific deviations from the grand mean. Intraclass correlations (ICCs) were calculated using the between and within covariances provided within the analysis output. These ICCs describe the proportion of variance found at the between-persons level for each item. Additional parameters were specified in the model that determine the difference between the unstandardized factor loadings at the between and within level. In addition to fitting an unconditional model examining between- and within-person measurement parameters for the stress factor, gender (dummy coded: male = 1, female = 0) was added as a covariate at the between-persons level (see Figure 2).^3^

#### Bayesian Estimation

*Mplus* 8.3 (Muthén & Muthén, 2017) uses Bayesian estimation for DSEM, which allows for a larger number of random effects than would be estimable using maximum likelihood (B. Muthén & Asparouhov, 2012). The Bayesian approach integrates prior information for each model parameter with the observed data to simulate a posterior distribution for each model parameter (Gelman et al., 2014; Lynch, 2007). The prior distribution includes information about the researcher’s hypothesis regarding parameter values and their confidence in the hypothesis (Lynch, 2007). *Mplus* uses very diffuse or uninformative priors by default, which contain little to no preexisting information regarding parameter values (Asparouhov & Muthén, 2021). As such, the simulated posterior parameter distributions are based almost entirely on the data, so results are very similar to what would be obtained using maximum likelihood estimation (Gelman et al., 2014; B. Muthén & Asparouhov, 2012).

Bayesian estimation in Mplus simulates posterior distributions via the Markov chain Monte Carlo (MCMC) algorithm, using a Gibbs sampler (Lynch, 2007). By default, *Mplus* utilizes two MCMC chains for a given number of iterations, discards the first half of the simulations (also known as the burn-in period), and determines convergence using the potential scale reduction (PSR), which is calculated using second half of the total iterations (Asparouhov & Muthén, 2021). The PSR criterion is computed for each parameter and represents the ratio of between chain variance across the MCMC chains to the variance within a chain. The closer the PSR criterion is to a value of one, the better the MCMC chains converged with one another, and if the PSR criterion is much larger than one, this indicates that the MCMC chains are instead diverging to different areas of the parameter space. Once convergence is obtained using the PSR criterion, it is recommended that the model be re-estimated using 3 to 4 times the number of iterations suggested by PSR to prevent “premature stoppage” (B. Muthén & Asparouhov, 2012). The median of the posterior distribution serves as the point estimate and a credible interval is calculated based on the upper and lower bounds of the posterior distribution based on predetermined cut-offs (e.g. 95% confidence interval [CI]; Lynch, 2007).

#### Summary of Analyses

The results of the current study are presented in stepwise fashion. First, the analyses from the measurement model are provided, including the intraclass correlations for each observed indicator, as well as the between and within *R*^2^ values and unstandardized factor loadings. This information provides insight into the noninvariance and reliability of each factor on the DASS-21 at the between and within levels. The *R*^2^ values represent the proportion of variance in each indicator that is attributable to the latent variable (i.e., true score variance; Nunnally & Bernstein, 1994; Raykov & Marcoulides, 2011), thus resulting in separate *R*^2^ values for each indicator at the between and within level. These values can be thought of as similar to standardized factor loadings and they are used in the present study to examine the reliability of each item between and within persons.

The second set of findings report on the structural results of the unconditional DSEM-CFA model, meaning that no covariates were added to the between level. Included here are the parameter estimates and credible intervals for the means of phi (φ) and innovation (ζ) as well as the variances for these and the between-persons latent stress (η*Str*^(b)^). These means and variances provide insight into the average level of carryover and innovation variance across all subjects, as well as how much carryover, innovation, and between-persons latent stress vary across persons. In addition, correlations between φ, ζ, and η*Str*^(b)^ are reported, which explain the relationships among the random parameters at the between-persons level. Finally, the results from the conditional model including gender are reported. These results illuminate the degree to which the random parameters (i.e., φ, ζ, and ηStr^(b)^) are predicted by gender.

## Results

### Measurement Model Results

Intraclass correlations (ICCs), factor loadings, *R*^2^ values, and difference tests for the measurement model parameters are provided in Table 1. The ICCs represent the proportion of total variance in each indicator that is found at the between level, and the *R*^2^ estimates describe the proportion of variance in that item that can be attributed to the true score, at each level. On the contrary, the unstandardized loadings represent the strength of the relationship between the item and the true score of the latent factor. Another way to conceptualize the difference between unstandardized factor loadings and *R*^2^ values is that unstandardized loadings indicate the level of sensitivity to changes in true score, whereas *R*^2^ values measure the reliability of the item.

**Table 1.** Between- and Within-Person Factor Loadings and Intraclass Correlations.

	Item Number	ICC	Within		Between		Difference
Factor Name			Unstd. Λ	*R* ^2^	Unstd. λ	*R* ^2^	
Depression	*Item 3*	.335	1.000	.339	1.000	.784	—
	*Item 5*	.491	.757	.199	1.000	.473	**.242 [.073, .431]**
	*Item 10*	.359	.979	.343	1.018	.768	.039 [−.105, .190]
	*Item 13*	.293	1.089	.317	.947	.689	**−.141 [−.284, .004]**
	*Item 16*	.295	1.098	.387	1.000	.843	−.098 [−.222, .026]
	*Item 17*	.407	1.004	.347	1.027	.611	.023 [−.141, .192]
	*Item 21*	.453	.703	.301	.709	.447	.006 [−.144, .156]
Anxiety	*Item 2*	.604	.761	.121	1.029	.378	**.267 [.015, .557]**
	*Item 4*	.408	1.177	.224	1.033	.636	−.144 [−.378, .118]
	*Item 7*	.364	1.288	.257	.953	.607	**−.335 [−.588, −.062]**
	*Item 9*	.507	.971	.130	1.215	.517	.245 [−.017, .533]
	*Item 15*	.377	.905	.188	.656	.425	**−.249 [−.434, −.044]**
	*Item 19*	.375	1.030	.221	.704	.438	**−.326 [−.533, −.103]**
	*Item 20*	.512	1.000	.180	1.000	.449	—
Stress	*Item 1*	.503	1.240	.357	1.227	.938	−.013 [−.184, .182]
	*Item 6*	.510	1.244	.363	1.224	.914	−.019 [−.195, .177]
	*Item 8*	.577	.928	.207	1.068	.547	.140 [−.045, .350]
	*Item 11*	.462	1.445	.445	1.207	.956	**−.237 [−.408, −.049]**
	*Item 12*	.592	.896	.200	1.111	.580	**.216 [.024, .429]**
	*Item 14*	.567	.797	.183	.900	.481	.103 [−.076, .293]
	*Item 18*	.563	1.000	.274	1.000	.586	—

*Note.* ICC = intraclass correlation. Bolded values indicate 95% credible intervals which do not contain zero.

To accurately examine invariance, one item must be selected as a marker to establish the scale for the latent variable by fixing the unstandardized factor loading for that item to 1 across both levels. The loadings for the remaining items are then estimated relative to the anchor item. If a noninvariant item is used as the anchor, the loadings for other items and thus the difference tests will be biased. In this study, an invariant item was selected using an adapted version of the triangle heuristic (Cheung & Rensvold, 1999). Invariant sets were identified using credible intervals for the loading difference tests and an invariant item was picked from the set to be used as an anchor item.

#### Reliability Results

The ICC for the indicators of depression ranged from .293 (Item 13 [“I felt down-hearted and blue.”]) to .491 (Item 5 [“I found it difficult to work up the initiative to do things.”]), suggesting that, for depression, more item variance was generally found at the within level than at the between level. The indicators for depression exhibited a fairly homogeneous range of *R*^2^ estimates at the within level, with most items’ within-person true depression score explaining 30% to 40% of the within-persons variance of that item. The only exception to this was Item 5, which had an *R*^2^ of only .199. The reliability for the indicators of between-persons depression was higher, with 44.7% (item 21 [“I felt that life was meaningless.”]) to 84.3% (item 16 [“I was unable to become enthusiastic about anything.”]) of the variance at the between level being explained by true scores. Most of the indicators of depression exhibited acceptable reliability at the between level, with only two items (Items 5 & 21) having *R*^2^ values below .6.

The ICCs for the indicators of anxiety ranged from .364 (Item 7 [“I experienced trembling [e.g., in the hands].]) to .604 (Item 2 [“I was aware of dryness of my mouth.”]), with all but one of the ICCs suggesting that more than half of the total item variance was found at the within level. The indicators for anxiety exhibited very low *R*^2^ estimates at the within level; for all items, the within-person true score of anxiety explained less than 30% of the within-persons variance. The reliability for the indicators of between-persons anxiety was higher, with 37.8% (Item 2) to 63.6% (Item 4 [“I experienced breathing difficulty [e.g., excessively rapid breathing, breathlessness in the absence of physical exertion]]”) of the variance at the between level being explained by true scores. Item 4 exhibited the highest reliability of the anxiety items at both the within and between levels, whereas Item 2 exhibited the lowest reliability at both levels.

The ICC for the indicators of stress ranged from .462 (Item 11) to .592 (Item 12), suggesting that an approximately equal amount of total item variance is found at the between and within levels. The indicators for stress exhibited a wide range of *R*^2^ estimates at the within level, with 18.3% (Item 14, “I was intolerant of anything that kept me from getting on with what I was doing”) to 44.5% (Item 11, “I found myself getting agitated”) of the within-persons variance being explained by true scores on latent within-persons stress. There is some variability in the within-persons reliability of these items, but overall, they do not exhibit adequate levels of reliability at the within level. The reliability for the indicators of between-persons stress is higher, with 48.1% (Item 14) to 95.6% (Item 11) of the variance at the between level being explained by true scores. Generally, the indicators of stress exhibit acceptable reliability at the between level. In fact, three of the seven indicators (Items 1 [“I found it hard to wind down,” 6 [“I tended to over-react to situations”], & 11) exhibit *R*^2^ estimates above .9 at the between level and these items are also the most reliable indicators of within-persons stress. On the contrary, Item 14 exhibits the worst reliability at both the between and within levels, at *R*^2^ = .481 and .183, respectively.

All three factors of the DASS-21 exhibited better reliability at the between level than they did at the within level. The stress factor had the highest *R*^2^ estimates at the between level, but at the within level, was more equally matched with the depression factor. The anxiety factor exhibited the lowest *R*^2^ estimates at both the between- and within-person levels across the three factors.

#### Invariance Results

The unstandardized factor loadings presented in Table 1 provide insights into the items’ sensitivity to changes in the true scores at each level. Using an invariant item as an anchor scales the indicators to a common metric across the levels and allows for direct comparison of factor loadings (Cheung & Rensvold, 1999; Mehta & Neale, 2005). An anchor item was identified for each factor of the DASS-21 and the unstandardized factor loadings of these items were set to 1 at both the within and between levels.

For five of seven items in the depression factor, posterior distributions supported invariance across the between and within levels. Items 5 and 13 were found to be noninvariant, meaning that they show different levels of sensitivity to true score changes at each level. Item 5 was found to be more sensitive to changes in the between-persons true score, which is congruent with its higher reliability at the between-persons level as well. Item 13 was found to be more sensitive to changes in the within-persons true score, despite having a higher *R*^2^ estimate at the between-persons level.

The same pattern was observed for the stress factor, with five of the seven posterior distributions supporting invariance across the between and within levels. Again, two items (11 & 12) were found to be noninvariant. Item 12 was found to be more sensitive to changes in the between-persons true score as well as being more reliable at the between level. Item 11 exhibited higher sensitivity to changes in the within-persons true score, even though it had a higher *R*^2^ estimate at the between-persons level.

The anxiety factor exhibited the most noninvariance, with only three of seven posterior distributions supporting invariance across the two levels. Items 2, 7, 15 (“I felt I was close to panic.), and 19 (“I was aware of the action of my heart in the absence of physical exertion [e.g., sense of heart rate increase, heart missing a beat]”) were found to be noninvariant across the within and between levels. Item 2 exhibited a higher sensitivity to changes in the between-persons true score, congruent with its higher reliability at that level. On the contrary, Items 7, 15, and 19 were all found to be more sensitive to changes in the within-persons true score, despite their very poor reliability at that level.

The posterior distributions for eight of the 21 items on the DASS-21 provided evidence for noninvariance across the within and between levels, with anxiety being disproportionately represented in these items. Of the noninvariant items, five were stronger indicators of within-persons differences, whereas three were more indicative of between-persons differences.

### Structural Model Results

#### Unconditional Model

Structural parameter results for the unconditional model can be found in Table 2. The posterior distributions for the phi (φ) and innovation (ζ) parameters provide support that these values are nonzero. These results suggest that an individual’s stress at time *t* is predicted by both their stress at the previous therapy session (one week prior) and by random innovations or disturbances between sessions. The random innovations reflect the various events and stressors that may have affected an individual’s stress level between measurement occasions and their level of sensitivity to those events. φ is positive for all three factors, indicating that the score at time *t –* 1 has a direct relationship with the score at time *t*.

**Table 2.** Unconditional Model Parameters.

Parameter	Depression	Anxiety	Stress
φ—*Mean*	0.454 [0.385, 0.519]	0.323 [0.249, 0.394]	0.341 [0.264, 0.417]
φ—*Var.*	0.072 [0.050, 0.102]	0.085 [0.057, 0.121]	0.080 [0.049, 0.116]
ζ—*Mean*	−3.263 [−3.485, −3.051]	−4.122 [−4.339, −3.899]	−3.164 [−3.373, −2.963]
ζ—*Var.*	2.350 [1.931, 2.871]	1.978 [1.622, 2.420]	1.591 [1.264, 1.988]
η^(b)^—*Var*.	.247 [.185, .323]	.174 [.119, .243]	.264 [.195, .348]
*r*_φ,ζ_	.255 [−.001, .479]	.506 [.249, .735]	−.156 [−.426, .135]
*r*_φ,η(*b*)_	.704 [.483, .868]	.602 [.349, .818]	.181 [−.073, .412]
*r*_ζ,η(*b*)_	.647 [.529, .745]	.837 [.755, .897]	.588 [.468, .688]

*Note.* η^(b)^ indicates the between-level latent variable. Parameter estimates for φ, ζ and η^(b)^ are unstandardized.

The correlations between φ and ζ for depression and anxiety are positive and the credible intervals do not contain zero, suggesting that for both factors, individuals with higher inertia or carryover from one session to the next also had higher random innovations or disturbances in their scores from week to week. For stress, the correlation between φ and ζ was negative but the posterior distribution provides little evidence that this result is credible.

The correlations between φ and between-persons true scores on all three factors are positive but only depression and anxiety evidence credible intervals that do not contain zero. Depression exhibits the strongest positive relationship, which indicates that individuals who have higher mean-level depression also tend to have high depressive inertia. In other words, their depression changes in a more consistent manner. Although this relationship is the same for anxiety and stress, it is slightly weaker. This relationship is weakest for the stress factor, with the credible interval just containing zero. This suggests that those with higher between-persons true scores for stress may have slightly higher carryover, but this result is not well supported by the posterior distribution.

Conversely, the positive correlations between between-persons true scores and ζ are supported by their posterior distributions for all three factors, suggesting that those with higher between-persons true scores were more likely to exhibit random disturbances in their scores from week to week, which could be due to an increased number/intensity of stressors or increased sensitivity to stressors that arise. This relationship was especially strong for the anxiety factor.

#### Conditional Model

Parameter results for the conditional model can be found in Table 3. The means and variances reported for φ and ζ now represent the intercepts and residual variances for these parameters after the model has been considered. Given that women were used as the reference groups on the dummy-coded covariate, these intercepts reflect the means for women. For example, the average φ for depression in White women is .428. There were few robust findings for the effects of gender on carryover, innovation, or between-persons true scores. There is evidence that between-persons stress and stress innovation variance do differ depending on gender. Specifically, the posterior distributions provide support that men exhibit lower mean-level stress, as well as less random disturbances in state stress, than women. There is some evidence that this same pattern exists in men within the anxiety factor, such that men exhibited lower mean-level anxiety and less random disturbances in their state anxiety.

**Table 3.** Conditional Model Parameters.

Parameter	Depression	Anxiety	Stress
φ—*Mean*	0.428 [0.342, 0.514]	0.316 [0.225, 0.408]	0.353 [0.258, 0.444]
φ—*Var.*	0.070 [.046, .101]	0.085 [0.052, 0.125]	0.085 [0.053, 0.125]
ζ—*Mean*	−3.100 [−3.362, −2.842]	−3.948 [−4.207, −3.679]	−2.943 [−3.176, −2.705]
ζ—*Var.*	2.336 [1.905, 2.862]	1.915 [1.560, 2.358]	1.441 [1.123, 1.834]
η^(b)^—*Var*.	.267 [.196, .358]	.176 [.118, .249]	.261 [.106, .349]
$\beta_{\phi \times \mathrm{male}}$	.123 [−.098, .335]	−.040 [−.274, .192]	−.099 [−.329, .138]
$\beta_{\zeta \times \mathrm{male}}$	−.086 [−.212, .046]	−.114 [−.247, .021]	**−.236 [−.366, −.097]**
$\beta_{\eta \left(b\right)\times \mathrm{male}}$	.028 [−.093, .154]	−.096 [−.217, .028]	**−.152 [−.267, −.036]**

*Note.* η^(b)^ indicates the between-level latent variable. Parameter estimates for φ, ζ, and η^(b)^ are unstandardized.

## Discussion

### Summary of Findings

The present study illustrated the application of DSEM to examine the reliability of an instrument to be used for longitudinal data collection. More specifically, a DSEM-CFA was applied to each factor of the DASS-21 in a sample of 333 individuals who sought therapy at a community mental health training clinic. Model results illuminated several aspects of psychometric and structural interest. Of the 21 items on the DASS, eight of them were found to be noninvariant, meaning that they have differing levels of sensitivity to true score change at the between and within levels. Anxiety was overrepresented among the noninvariant items, with half of all noninvariant items located in the anxiety factor. Generally, the noninvariant items were stronger indicators of within-persons true score rather than between-persons true score. It is important to note that an item with a higher unstandardized loading at the within level is not necessarily more reliable at the within level. This is because the unstandardized loading is a regression coefficient reflecting the extent to which changes in the latent factor predict changes in the item. On the contrary, reliability is reflected through the *R*^2^ values, which demonstrate the proportion of each item’s variance that is explained by the latent factor. The DASS-21 exhibits fairly good reliability at the between-level but poor reliability at the within-level, indicating that it is more effective at differentiating between clients with higher or lower levels of symptomology than it is at detecting meaningful change over time. This is inconsistent with previous research on the DASS, which has reported that the instrument is adequately sensitive to change, although some research has noted that the DASS is less sensitive to change than other measures of the same symptomology (Ng et al., 2007; Page et al., 2007). Of the three factors, anxiety appears to be the least reliable within the DASS-21 at both the within and between levels.

The innovation variance for stress was higher than that of depression and anxiety, although the current study was unable to test these differences directly. The carryover was strongest for depression, with the stress and anxiety factors exhibiting lower and more similar carryover values. Consistent with previous research, the correlation between innovation and between-level true score depression was positive and very strong, suggesting that individuals with higher levels of depression overall were exhibiting more random disturbance in their depression between sessions (Wichers et al., 2009). It is important to remember that higher innovation variance could indicate more external events that affect the state score or a higher sensitivity to those events (Hamaker et al., 2018). Anxiety and stress also exhibited positive correlations between between-level true scores and innovation variance. This correlation was strongest for anxiety. For the depression and anxiety factors, carryover was positively correlated with both innovation variance and between-level true scores, meaning that individuals with higher inertia in scores were also more depressed or anxious overall, as well as exhibiting more random variations in scores from session to session. Somewhat surprisingly, there were few robust effects gender across the three factors. The best supported findings suggested that men exhibited less stress overall (i.e., mean-level stress) and were also less likely to exhibit random variations in stress from session to session. This could mean that men are less likely to experience external events that affect their stress level, or more likely, their stress levels are less sensitive to external events. A similar pattern emerged for men in the anxiety factor, but the evidence for this was not as strong.

### Implications and Future Directions

Results from any longitudinal study are questionable unless the instrument being used to measure the outcomes have been properly validated for use over several time points. This study is an example of how an instrument with strong evidence of between-persons reliability may not be reliably capturing individual change over time. The DSEM-CFA offers an opportunity to examine the instruments we currently use for longitudinal research for their between- and within-person reliability. In addition, the results from this study call into question the instruments clinical researchers use within randomized controlled trials (RCTs) to establish evidence for existing therapy modalities. As RCTs require at least two observed data points to measure treatment effects between at least two groups of participants, it is important to examine the reliability of the instruments chosen for comparing groups (between-persons differences) and measuring treatment effects (within-persons change). Until reliability has been adequately established, the resulting conclusions from longitudinal studies will remain questionable. The DSEM-CFA provides the most thorough examination of between- and within-persons reliability that is currently available to clinical researchers.

There are several facets of the DSEM-CFA still to be explored. For example, simulation studies exploring different aspects of power in a DSEM-CFA would be useful to establish what criteria of sample size and time points must be met for a well-powered study of between and within reliability. In addition, as the DSEM-CFA is already a very complex and detailed model, future studies could explore potential pathways for including the complete factor structure of an instrument, rather than only examining one factor at a time. Clinical researchers would also benefit from learning and applying the DSEM-CFA for an instrument they desire to use across multiple measurement occasions. If an instrument currently being used for RCTs or other longitudinal research is found to be unreliable within persons, the results of these studies could be questionable. Future studies seeking to validate an instrument for use in longitudinal research should consider utilizing the DSEM-CFA, as it presents one of the most detailed and unbiased methods for examining reliability both between and within persons while accounting for the autoregressive relationship between measurement occasions.

The results of this study have specific implications for the use of the DASS-21. Although previous research has reported that the DASS is a good tool for longitudinal research (Ng et al., 2007), the current study provides opposing evidence. The DASS-21 exhibited acceptable reliability at the between-persons level, consistent with the many studies that have evidenced this with cross-sectional samples (Clara et al., 2001; Crawford & Henry, 2003; Henry & Crawford, 2005; Page et al., 2007). However, the within-persons reliability was very poor. This supports the use of the DASS-21 as a screener or intake tool to get a snapshot of their depression, anxiety, or stress at a given time. The DASS-21 is a good tool for comparing individuals with each other, meaning that it is adequate at differentiating between someone who is mildly depressed and someone who is severely depressed at single measurement occasion. This is what the DASS was originally designed to do and the evidence from this study supports its use in this way (Lovibond & Lovibond, 1995).

### Limitations

This study has several limitations to consider. First, the level of detail that the model provides restricted the number of factors that could be estimated at once. Therefore, each factor of the DASS-21 was estimated separately. Given the overlapping nature of depression and anxiety (Clark & Watson, 1991), modeling each factor individually does not allow for a complete representation of the constructs of interest. The models explored in the current study did not allow for any examination of the relationships between depression, anxiety, and stress at the state and trait level. The DSEM-CFA is still an emerging procedure, so it is possible that solutions will arise that allow for multiple factors to be estimated despite the extreme complexity. The lack of previous research on the DSEM-CFA, although presenting an opportunity for the current study, also provides more potential for errors or unknown biases. The methodological nuances of the model have yet to be explored and it is expected that over time the method will become more advanced and precise.

Within the current study, the factor indicators were treated as continuous variables and it could be argued that treating the indicators as ordinal response variables (i.e., an item response theory [IRT] model) would provide more detailed insight into how participants respond to individual items. However, convergence of the present model with ordinal indicators would have required a larger sample size and more measurement occasions (Forero et al., 2009). In addition, Schultzberg and Muthén (2018) suggest that at least 10 time points are required for DSEM analyses. The sample in the current study had an average of 10 observed data points for each individual, with significant variation between individuals. It is unclear from previous research whether an average of 10 measurement occasions is sufficient for DSEM analyses versus requiring a minimum of 10 data points for each participant. Therefore, the current study may be lacking somewhat in power because previous research did not clearly address the DSEM-CFA or situations in which number of observations vary across individuals. However, Schultzberg and Muthén (2018) also evidence that large sample sizes (i.e., *N* ≥ 200) allow for a smaller number of observations (i.e., T = 10), but it is unknown how this may apply to a DSEM-CFA. It is also dependent on how effective the MCMC algorithm is at handling missing data. Although Asparouhov and colleagues (2018) suggest that DSEM can handle up to 95% missingness with 10 measurement occasions, it is unclear how this might apply to a DSEM-CFA or values that are missing not at random, as was the case in the current research. It would be beneficial to rerun the present analyses with more data at a future time.

The sample in this study was involved in active treatment, and thus, systematic trends over time may be present in the data. Given that DSEM models assume stationarity, this limitation may introduce some bias into the parameter estimates. However, this bias is likely to be minimal, as the sample had received an average of almost 60 therapy sessions and systematic change is less likely than it would be early in treatment. It is possible to incorporate additional levels of random effects, such as including time as its own level (i.e., cross-classified growth model). However, aspects of the present study’s design did not allow for the evaluation of a cross-classified model at this time.^4^ Future studies should attempt to identify data sets that would support a cross-classified approach.

The sample in the current study was a relatively diverse, naturalistic sample, which in many ways is considered a strength. However, the current study was unable to examine differences in factor loadings or reliability as a result of differences in race/ethnicity or gender. The reliability within persons may be stronger for specific groups, but the results of the present research are not able to provide insight into this question. Similarly, within-person noninvariance was not able to be tested in this study because of the assumption of this model that the within-person structure remains invariant over time. Although techniques utilizing cross-classified variance of the DSEM model are emerging (McNeish et al., 2021), the number of measurement occasions used in the present data, along with the complexity of the measurement model, did not allow for the investigation of noninvariance over time. Similarly, transgender and nonbinary individuals were dropped from the conditional model because there were too few individuals within these categories. As a result, this study was unable to identify differences that may exist within the structural parameters for gender identities outside of ciswomen and cismen.

## Conclusion

In recent years, significant progress has been made in examining reliability within and between persons while accounting for the autoregressive relationship between measurement occasions (Asparouhov et al., 2018). This study applied a newly created psychometric method, DSEM-CFA, to each subscale of a commonly used instrument, the DASS-21. The results revealed that the DASS-21 exhibits acceptable reliability between persons, but substandard reliability within persons. The current study also explored other facets of the DSEM-CFA, such as carryover and innovation variance, and their random effects. Results suggested that state scores were influenced both by the previous session, 1 week prior, and random disturbances or events that may have occurred between sessions. The DSEM-CFA model in this study highlights the importance of validating instruments within persons. Specifically, the model presented here allows for one unified approach to between- and within-persons reliability while accounting for and directly examining autoregression, thus reducing bias and increasing accuracy and nuance. There are currently several instruments and tools being used to measure symptoms over time that have yet to be adequately validated for that use. Although there are several limitations with the model currently, many of these could be solved for after exploration through simulation studies or other methodological publications. Future studies would benefit from utilizing the DSEM-CFA to examine reliability, noninvariance, autoregression, and covariate effects within the instruments being used for longitudinal research.

## Notes

1. The majority of the participants had been receiving therapy prior to introducing the tablet administration and in total had been seen in therapy an average of 58.19 (*SD* = 70.8) sessions.

2. The MPlus syntax and output files can be viewed at: https://osf.io/cts6g/?view_only=b40ad5a5dda94370a8b64c7c0346228b.

3. All others (*n* = 14) were removed for the conditional analysis since the sample sizes for nonbinary and transgender individuals were too small.

4. A cross-classified model was attempted with these data. However, in order for this model to be estimable, Mplus requires the time variable to start at 1 for all people. To construct the time variable so that it meets this requirement would violate another requirement that the time variable is aligned across people (i.e., T = 1 means the same thing everyone).
